# Perfect Hamming code with a hash table for faster genome mapping

**DOI:** 10.1186/1471-2164-12-S3-S8

**Published:** 2011-11-30

**Authors:** Yoichi Takenaka, Shigeto Seno, Hideo Matsuda

**Affiliations:** 1 Department of Bioinformatic Engineering, Graduate School of Information Science and Technology, Osaka Univesity, 1-5 Yamadaoka, Suita, Osaka, Japan

## Abstract

**Background:**

With the advent of next-generation sequencers, the growing demands to map short DNA sequences to a genome have promoted the development of fast algorithms and tools. The tools commonly used today are based on either a hash table or the suffix array/Burrow–Wheeler transform. These algorithms are the best suited to finding the genome position of exactly matching short reads. However, they have limited capacity to handle the mismatches. To find n-mismatches, they requires *O*(2*^n^*) times the computation time of exact matches. Therefore, acceleration techniques are required.

**Results:**

We propose a hash-based method for genome mapping that reduces the number of hash references for finding mismatches without increasing the size of the hash table. The method regards DNA subsequences as words on Galois extension field *GF*(2^2^) and each word is encoded to a code word of a perfect Hamming code. The perfect Hamming code defines equivalence classes of DNA subsequences. Each equivalence class includes subsequence whose corresponding words on *GF*(2^2^) are encoded to a corresponding code word. The code word is used as a hash key to store these subsequences in a hash table. Specifically, it reduces by about 70% the number of hash keys necessary for searching the genome positions of all 2-mismatches of 21-base-long DNA subsequence.

**Conclusions:**

The paper shows perfect hamming code can reduce the number of hash references for hash-based genome mapping. As the computation time to calculate code words is far shorter than a hash reference, our method is effective to reduce the computation time to map short DNA sequences to genome. The amount of data that DNA sequencers generate continues to increase and more accurate genome mappings are required. Thus our method will be a key technology to develop faster genome mapping software.

## Background

The history of bioinformatics has been dominated by the search for faster sequence alignment methods. Beginning with dynamic programming for protein and genome sequence alignment, many algorithms have been proposed. Hash tables are used in the series of FASTA programs [1], which calculate approximate alignments in shorter times than dynamic programming can. BLAST tools, using automatons in their algorithms, are the most famous and most used alignment tools [2]. These tools are fast enough to align expression sequence tags generated by capillary electrophoresis-based DNA sequencers to target genomes.

The emergence of next-generation sequencing technology has changed the demands for alignment speed. A so-called next-generation sequencer can read far more base pairs than a conventional sequencer: more than two billion short DNA sequences in a single run. For such a large number of the sequences, BLAST tools are too slow to map the sequences to target genomes. Therefore, researchers have called for a faster approach that is focused on mapping short fragments.

To meet this demand, more than 25 software programs designed for mapping short DNA sequences onto genomes have been developed. These are classified into two categories according to their algorithms, which are either hash-based or suffix array/Burrow–Wheeler transition (BWT)-based [3], MAQ [4] and SOAPv1 [5] are two hash-based algorithms. The former indexes short DNA sequences and the latter indexes genome sequences. mrsFAST is the one of the newest algorithms that indexes both the short DNA sequences and the genome sequences [6]. The first genome-mapping algorithm based on suffix arrays was proposed in 2002 [7] and implemented as vmatch. Currently, a BWT-based algorithm is the fastest and is used in four tools: bowtie [8], BWA [9], SOAPv2 [10], and segemehl [11].

These algorithms are effective for mapping short sequences to genome positions of perfect matches and one-base mismatches, but are inefficient for mapping to positions for two or more-base mismatches. In general, they require *O*(2*^n^*) computation time to calculate *n*-base mismatches. But genome mapping that allows only one-base mismatches is inadequate. In practice, 20 to 40% of short sequences cannot be mapped to the genome. Therefore, “wet” researchers require faster algorithms for mapping short sequences to genome positions with two or more-base mismatches. In this paper, we propose a method that can accelerate hash-based genome mapping by reducing the number of hash references without increasing the size of the hash table.

In the proposed method, DNA subsequences are divided into equivalence classes by using a perfect Hamming code. Each equivalence class includes subsequences whose corresponding words on *GF*(2^2^) are encoded to the corresponding code word of the perfect Hamming code. The code word is used as a hash key to store these subsequences in a hash table. A perfect Hamming code is a special case of a Hamming code, known in the field of coding theory [12], that satisfies the Hamming bound with equality. Perfect Hamming codes have been applied to n-gram analysis of genome sequence [13] and multiple alignment [14].

Hash-based genome-mapping algorithms use hash tables. A hash table is an array indexed by hash values generated from hash keys. Thus, a hash table is an implementation of an associative array. There are two methods for mapping short reads onto genomes using hash tables. One is to store subsequences of the genome and their positions in a hash table and the other is to store subsequences of short reads. As there is no essential difference between their hash usages, we use the former method for the following explanation.

The hash-based methods prepare a hash table whose keys and values represent subsequences of length *l* cut from a target genome and the subsequence genome positions, respectively. To map a short sequence to the genome, a sequence is cut into lengths *l*, and these are used as keys to refer to the hash table. The methods can find the genome position of a perfect match if the hash table returns at least one entry. In general, when the lengths of short sequences are longer than the length of the hash key, the methods expand the area of alignment from the genome position. The differences between methods are how and when they refer to the hash table.

There are three methods to find the n-mismatch genome positions of a subsequence of length *l* with the hash table.

1. Refer to all n-mismatch subsequences.

Prepare a hash table whose key length is *l*, and use the subsequence and its n-mismatch subsequences as keys to refer to the table. It requires  hash references to find all the n-mismatch genome positions.

2. Store n-mismatch positions in the hash table.

For each position of the subsequence of the genome, store the position  times. The hash keys are the subsequence and its n-mismatch subsequences.

3. Use pigeonhole principle; combine hash table and another method.

Generate a hash table whose key length is ⌊*l*/*n*⌋. After getting the perfect-match genome position of length ⌊*l*/*n*⌋ by referring to the hash table, find n-mismatch sequences by another method, such as dynamic programming or BWT.

**Figure 1:**
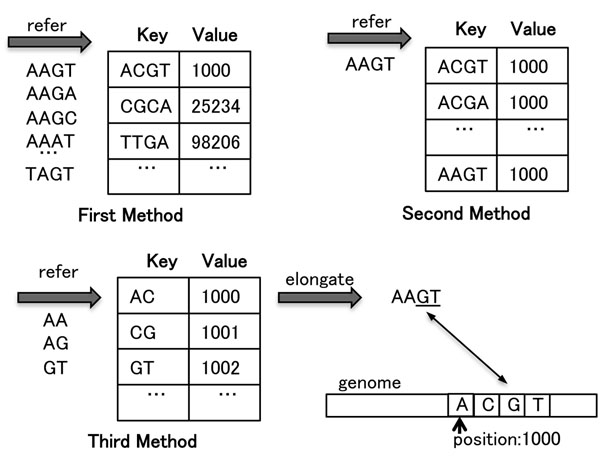
**Hash tables for three methods.** Three methods to find genome positions of 1-mismatch from the subsequence AAGT. Genome position 1000 is ACGT, which is the 1-mismatch of the subsequence. The first method refers to the hash table 16 times. The second method refers to the table just once, but the table is 16-fold larger. The third method refers to the table three times. After getting position 1002 from the hash table, the method elongates the alignment toward the front of the sequence.

Figure 1 shows examples of the three methods.

These methods are effective when *l* is small and *n* equals 1. But they are difficult to use when *n* is 2 or more because the number of 2-mismatch sequences of length *l* is *l*(*l* – 1)/2 ∗ 9 and that of 3-mismatches is *l*(*l* – 1)(*l* – 2)/2 ∗ 9. The first and second methods require too many hash references and too big a hash table, respectively. The third method is the best, but as *n* becomes larger, the ability to narrow the genome position down becomes weaker, and so the load of the post process to find n-mismatch sequences increases. To overcome these difficulties and improve the effectiveness of using hash tables for genome mapping, technical breakthroughs are needed.

We propose a method to reduce the number of hash references to find the genome positions of 2 or more mismatches without enlarging the size of the hash table. To realize the method, 4-ary perfect Hamming code is used.

## Results

### Perfect Hamming codes as hash keys

#### Idea

**Figure 2:**
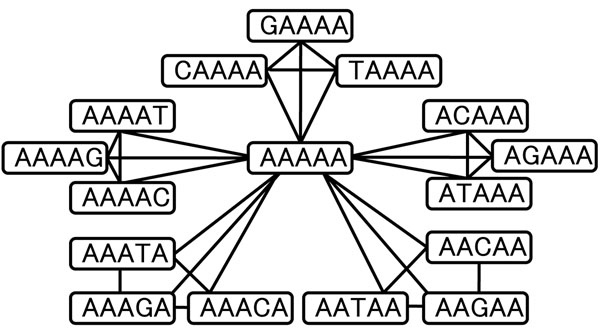
**Relationship among 16 subsequences.** Graphical depiction of subsequence “AAAAA” and 15 adjacent subsequences. Each node describes a subsequence and each edge indicates that the terminal nodes are of one nucleotide difference. The 15 subsequences are divided into five groups according to the position of the different nucleotide.

We first describe the main idea of the proposed method. We define a graph whose nodes are all the subsequences of length *l* and edges are between pairs of subsequences of one nucleotide difference. The graph has 4*^l^* nodes and each node has 3*l* edges. Assume we divide the 4*^l^* nodes of the graph into 4*^l^*/(3*l* + 1) equivalence classes such that each class has a center node and 3*l* adjacent nodes. Then, we use the center node as the hash key to store the genome positions of 3*l* + 1 subsequences in a hash table. For example, Figure 2 shows a part of the graph of length 5. The node “AAAAA” has 15 adjacent nodes. These comprise an equivalence class with subsequence“AAAAA” at its center. To store “ACAAA”, which belongs to the equivalence class, the center word “AAAAA” is used as a hash key.

The features of this hash table are as follows: (1) The number of entries in the hash table does not increase because each subsequence is stored only once. (2) Using this hash table, we can reduce the number of hash references to find the genome positions of subsequences of 1 or more-mismatches.

**Figure 3:**
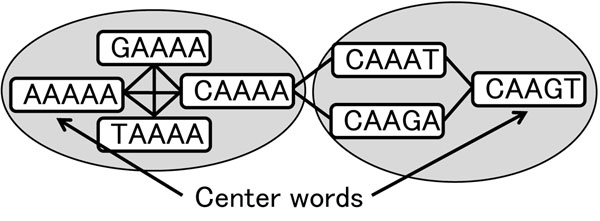
**1-mismatch sequences of a non-code word sequence, “CAAAA”**. Idea behind finding 1-mismatch sequences of the sequence “CAAAA”. The two circles indicate the equivalence classes. The sequence “CAAAA” belongs to an equivalence class whose center is “AAAAA” that holds 3 of the 15 1-mismatch sequences. Two of the other sequences, “CAAAT” and “CAAGA”, belong to a equivalence class whose center is “CAAGT”.

We explain the concept how to reduce the number of hash references to find 1-mismatches by using an example. Let the length of subsequence be 5, the hash table be as described above, and *s* be a subsequence for which we want to find the entries of 1-mismatch. There are 15 1-mismatch subsequences to be referred to. When *s* is the center subsequence of the equivalence class, such as “AAAAA” in Figure 2, using *s* as the hash key, one can find all the genome positions of 1-mismatch subsequences. When *s* is not the center subsequence of its equivalence class, seven hash keys are needed. One hash key is the center subsequence of its equivalence class. This leads to 3 of the 15 1-mismatch subsequences. The rest of 12 subsequences are members of six different equivalence classes, and so the center subsequence of these classes are used as the other six keys. Figure 3 shows the equivalence class that *s*=“CAAAA” belongs to and an equivalence class containing the two adjacent subsequences “CAAAT” and “CAAGA”. Because the proportion of center words (nodes) to total words is 1/16, the expected number of hash references is 1/16 ∗ 1 + 15/16 ∗ 7 = 6.625, which is 41.4% of the number of exact and 1-mismatch subsequences.

The requirements for the establishment of the equivalence classes need to be determined. At a minimum, the length of the subsequence *l* must satisfy the condition:(1)

This shows that 3*l* + 1 must be a power of 4. For example, *l* = 5 and 21 satisfies the condition.

It is not clear that the above equation is a sufficient condition for constructing equivalence classes. Even if it is, two problems still remain; how to construct the equivalence classes and how to calculate the center words from a given subsequence. Perfect Hamming codes provide solutions to both these problems.

#### Perfect Hamming code

A perfect Hamming code (PHC) is a Hamming code that satisfies the equation of the Hamming bound,(2)

where *C* is a set of *q*–ary block code of length *n*, *d* is the minimum Hamming distance between code words, and . In the PHC method, all the received words are classified into a code word or a 1-bit error. In other words, all the words are decoded to code words whose Hamming distance is 0 or 1.

The condition for a *q*-ary (*n*, *k*)-Hamming code be a perfect code is *n* = *q^k^*/(*q* – 1). In the case of a 4-ary code, (5,3)-Hamming code and (21,18)-Hamming code are perfect. Their parity-check matrixes are shown in equations (3) and (4), respectively.(3)(4)

where (0, 1, *α*, *α*^2^) are the elements in the Galois field GF(2^2^). To get a code word *x* from received word *z*, the syndrome *s* = (*z* – *x*)*H^T^* is used to determine the error vector *e* = (*z* – *x*) [12]. If the syndrome *s* is not zero, the column of *H* that is equal to or constant factor of *s* indicates the error position of *z*. The addition and multiplication tables are shown in Table 1.

**Table 1:** Addition and multiplication on *GF*(2^2^)

+	0	1	*α*	*α*^2^		×	0	1	*α*	*α*^2^
		
0	0	1	*α*	*α*^2^		0	0	0	0	0
		
1	1	0	*α*^2^	*α*		1	0	1	*α*	*α*^2^
		
*α*	*α*	*α*^2^	0	1		*α*	0	*α*	*α*^2^	1
		
*α*^2^	*α*^2^	*α*	1	0		*α*^2^	0	*α*^2^	1	*α*

The code word is calculated from a received word as follows.

1. Calculate the syndrome *s*.

2. If the syndrome *s* is zero, then the recieved word is the code word.

3. Find a column *c* of parity-check matrix that is a constant factor *t* of the syndrome.

4. Subtract *t* from the column *c* of the received word and the result is the code word.

For example, assume the word *z* = (*αααα*^2^0) is received. The code word of *z* is calculated as follows. The syndrome *s* of *z* is:

As *s^T^* is equal to *α*^2^ × (1 *α*), which is *α*^2^ times the fourth column of *H*, subtract (000*α*^2^0) from z:

The code word of *z* is (*ααα*00).

The (*n*, *k*)-Hamming codes are composed of the information digits and the check digits. The information digits are *k* arbitrary digits of the code and the other *n* – *k* digits are the check digits. The generator matrix *G* can reproduce check digits from information digits. Therefore, code words can be uniquely represented by the *k* digits. In this paper, we call these representations short codes.

#### PHC and DNA subsequence

DNA sequences are composed of four nucleotides, adenine, cytosine, guanine and thymine. Let these correspond one-to-one to the elements of Galois field *GF*(2^2^). Then, DNA sequences correspond to words on the Galois field. Without loss of generality, let (A,C,G,T) correspond to (0,1, *α*, *α*^2^). The sequence “GGGTA” is expressed as the word (*αααα*^2^0), and the word (10000) represents the DNA sequence “CAAAA”.

This correspondence relationship and the PHC enables us to build the equivalence classes described in Section *Idea*. Each equivalence class is composed of a DNA subsequence that corresponds to a PHC code word and DNA subsequences whose corresponding words are error-corrected to the code word. Figure 2 shows an equivalence class. The DNA subsequence “AAAAA” corresponds to word (00000) on *GF*(2^2^) and is a code word of 4-ary (5-3)-PHC. From the properties of PHC, All the words whose Hamming distances from the code word (00000) are 1 are error-corrected to the code word, and they are adjacent nodes of “AAAAA”. Additional File 1 shows correspondence table of 5-mer subsequences and code words of 4-ary (5,3)-PHC. In the following, we regard DNA subsequences and their corresponding codes on the Galois field as equivalent (i.e., aliases).

### Algorithms

We propose a hash table for genome mapping whose hash keys are code words of PHC. Then we show its use and efficiency in finding genome positions of n-mismatches and n-gaps. Following is a description of the notation used in this section.

#### Preparing the hash table

There are two ways to construct hash tables for mapping short DNA sequences onto a genome. One uses subsequences of the genome as hash keys to store their genome positions in a hash table. And another uses subsequences of short DNA sequence as the hash key. Because both of these use DNA subsequences as hash keys, our method can be applied to either. In the following, we use the former in the explanation.

**Figure 4:**
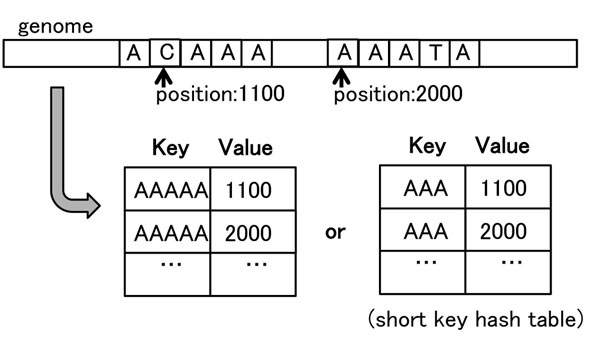
**Proposed hash table.** Entries of proposed hash tables. Subsequence “AAAAA” is used as the key for storing the genome positions of “ACAAA” and “AAATA” because“AAAAA” is the center of the equivalence class that “ACAA” and“AAAT” belong to. The left hash table uses the center subsequence itself as a hash key. The right one uses the short code of the center subsequence as a hash key, where the short code is the information digits of the code. Short codes are described in Section *Perfect Hamming Code*.

The hash table of our method uses the representative subsequence of the equivalence classes as hash keys. The representative is the code word on PHC. Without loss of generality, the information digits of the code words can be used as the hash key. Given an (*n*, *k*) – PHC on *G*(2^2^), let *S* be a set of subsequences of the genome with length *l* = *n*. The genome positions of a subsequence *s* ∈ *S* are stored in the hash table along with a hash key *c*(*s*) or its information digits *i*(*c*(*s*)). Figure 4 shows the use of hash tables.

#### Searching for n-mismatches

In this section we describe how to find genome positions of 1- and 2-mismatch subsequences of a given subsequence *s* given the hash table prepared as described in section *Preparing the hash table*. The efficiency of the method is also described.

Let *s* be a DNA subsequence. The set of hash keys *K* required to refer to all the entries of perfect match and 1– to *n*–mismatches is naturally expressed as follows.

The number of keys |*K_n_*(*s*)| is less than the number of subsequences to be referred to, . Specifically, when the length of *s* is 5 and 21, the expected number of keys for 1-mismatch *K*_1_(*s*) are 6.625 and 30.25, respectively. As the number of subsequences of perfect and 1-mismatch are 1 + _5_*C*_1_ × 3, the method reduces the number of hash keys to 41.4% (= 6.625/16) and 47.7% (= 30.25/64), respectively. We summarize these values in the “ratio” column of Table 2. In the following, we analyze the properties of the hash keys *K*_1_(*s*) and *K*_2_(*s*).

**Table 2:** Summary of our methods for lengths 5, 21, and 10 to refer to 1- and 2-mismatch and 1- and 2-gap sequences

length	condition	#keys	#words	ratio	*f*(*s*, *K*) when *s* = *c*(*s*)	*f*(*s*, *K*) when c ≠ *c*(*s*)
5	1-mismatch	6.625	16	41.4%	1 + 15*x*	1 + 15*x* + 42*x*^2^ + 54*x*^3^
	2-mismatches	27.25	106	25.7%	1 + 15 + 90*x*^2^ + 210*x*^3^ + 180*x*^4^	1 + 15 + 90*x*^2^ + 170*x*^3^ + 156*x*^4^
	1-gap	3.25	4	81.3%	4 + 12*x*	4 + 60*x*
	2-gaps	10	16	62.5%	16 + 36*x* + 108*x*^2^	– ∗^1^

21	1-mismatch	30.53	64	47.7%	1 + 63*x*	1 + 63*x* + 210*x*^2^ + 1710*x*^3^
	2-mismatches	611.31	1954	31.3%	1 + 63*x* + 1890*x*^2^ + 4410*x*^3^ + 34020*x*^4^	1 + 63*x* + 1890*x*^2^ + 5650*x*^3^ + 31500*x*^4^
	1-gap	3.81	4	95.3%	4 + 60*x*	4 + 252*x*
	2-gaps	13.87	16	86.7%	16 + 84*x* + 540*x*^2^	16 + 48*x* + 960*x*^2^

10: Serialize	1-mismatch	12.25	31	39.5%	1 + 30*x* + 225*x*^2^	1 + 30*x* + 170*x*^2^ + 538*x*^3^ + 1089*x*^4^ + 1620*x*^5^ ∗^2^
10: Parallelize	1-mismatch	13.25	31	44.1%	1 + 30*x*	1 + 30*x* + 84*x*^2^ + 108*x*^3^ ∗^3^

∗^1^ :*s* always includes one code word. ∗^2^: neither the first half nor the second half are code words. The reference formula when one of the two halves is a code word is 1 + 30*x*^2^ + 267*x*^2^ + 684*x*^3^ + 810*x*^4^. ∗^3^: neither the first half or second half are code words. The reference formula when one of the two halves is a code word is 1 + 30*x*^2^ + 42*x*^2^ + 54*x*^3^.

First, we analyze *K*_1_(*s*) for *s* of length 5. The subsequence *s* is classified into two cases according to whether *s* is a code word on PHC.

Case 1: *s* is a code word. As *s* is the code word, all the 1-mismatch words of *s* are decoded to *s* by PHC. That is to say, they belong to the same equivalence class *E*(*s*). Therefore, *s* is used as a hash key and it can refer to all the 1-mismatch subsequences.

**Figure 5:**
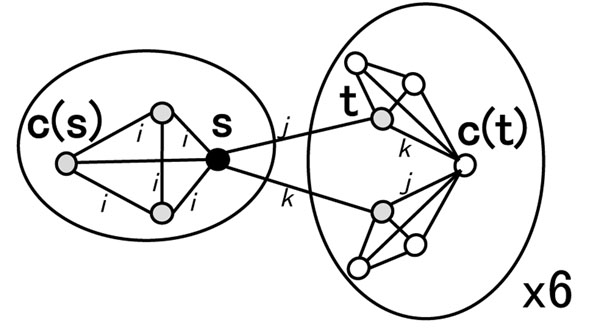
**Fifteen 1-mismatch subsequences of *s* when *s* is not a code word**. Nodes represent subsequences and edges indicate a Hamming distance between two nodes of 1, namely, the relation of 1-mismatch. Edge labels indicate the position of the different digit (nucleotide). *s* belongs to a equivalence class of *c*(*s*), which also contains three other words. The other 12 words belong to six equivalence classes.

Case 2: *s* is not a code word. Figure 5 shows the fifteen 1-mismatch subsequences. As *s* is not a code word, the equivalence class *E*(*s*) has three 1-mismatch subsequence of *s*. One of these three is the code word *c*(*s*). These three differ from *s* at the same digit. Assume *s*= “AAAAG”. Then, the code word *c*(*s*) is “AAAAA”, and The words “AAAAC” and “AAAAT” belong to same equivalence class, E(“AAAAG”). These differ at the fifth digit. The hash key *c*(*s*) can refer to four sequences at once.

The rest of 12 words belong to six equivalence classes. Assume that word *t* differs at *j*-th digit. *t* is not a code word because *d_H_*(*t*, *c*(*s*)) = 2 and the distance between code words must be more than 3. The code word *c*(*t*) and *t* differ at the *k*-th digit, where *k* ≠ *i*, *j*. There is a word *u* that differs from *s* at the *k*-th digit and *c*(*t*) at the *j*-th digit. Because *c*(*t*) belongs to the equivalence class *E*(*t*), use *c*(*t*) as a hash key and two words *t* and *u* can be referred to. Finally, the number of keys *K*_1_(*s*) to refer to all the 1-mismatch subsequences is seven.

Because the proportions of Case 1 and Case 2 are respectively 1/16 and 15/16, the expected number of keys in *K*_1_(*s*) is 6.625 (1 ∗ 1/16 + 7 ∗ 15/16).

Next, we show an algorithm to calculate the set of hash keys *K*_1_(*s*).

Input : s

*Output : K*_1_(*s*) *: a set of hash keys*

1. *K*_1_(*s*) *← c* (*s*)

2. *s* := *c* (*s*) and return (*K*_1_ (*s*))

3. for *t* in *N*_1_(*s*) add *c*(*t*) to *K*_1_(*s*)

4. return (*K*_1_(*s*))

The algorithm calculates code words 16 times when *s* is not a code word, where the number of the hash keys is seven. There appears to be redundancy. There are various ways of reducing the computation time of the code words, but it is better not to use complicated algorithms. The calculation of code words is fast because the operations + and × on *G*(2^2^) can be calculated as binary operations.

By using the set of hash keys *K*_1_(*s*), not only the entries of *s* and all the 1-mismatch subsequences are referred to, but also the other entries are. For example, when *s* is not a code word, 12 subsequences of 2-mismatch belongs to the same equivalence class *E*(*s*). Therefore, the hash key *c* (*s*) refers to these subsequences. To analyze the properties of these subsequences, we define a reference formula:

where *s* is a subsequence, *K* is a set of hash keys, and the coefficient *a* represents the number of subsequences that are referred to by *K* and whose distance from *s* is *b*.

For Case 1, because *s* is the code word, all the 1-mismatch subsequences belong to *E*(*s*). Therefore, the reference formula for Case 1 is:

For Case 2, there are 7 hash keys in *K*_1_(*s*). Because the equivalence class of *c* (*s*) has 12 2-mismatch subsequences, the reference formula of *c* (*s*) is:

Each of the equivalence classes of the other hash keys has two 1-mismatch sequences, five 2-mismatch sequences and nine 3-mismatch sequences. The reference formula of *K*_1_(*s*) – {*c*(*s*)} is:

Finally, the reference formula for the Case 2 is:

The reference formula shows the proposed method searches many 2- and 3-mismatch sequences. We discuss this feature in Section *Discussion*.

The above algorithm and analysis can be applied to the word length 21. The numbers of hash keys are 1 and 31 for Case 1 and Case 2, respectively. Using the rate of occurrences of Case 1 and Case 2, 1/64 and 63/64, respectively, the expected number of hash keys is 30.53. The reference formulas are shows in Table 2.

**Figure 6:**
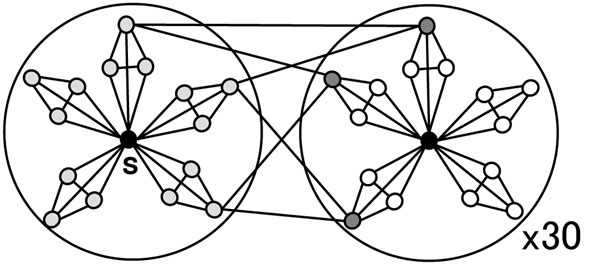
**Two-mismatch subsequences of *s* when *s* is a code word**. Dark-gray nodes are 2-mismatches whose Hamming distance from *s* is 2. Each belongs to an equivalence class that has other two 2-mismatche subsequences.

**Figure 7:**
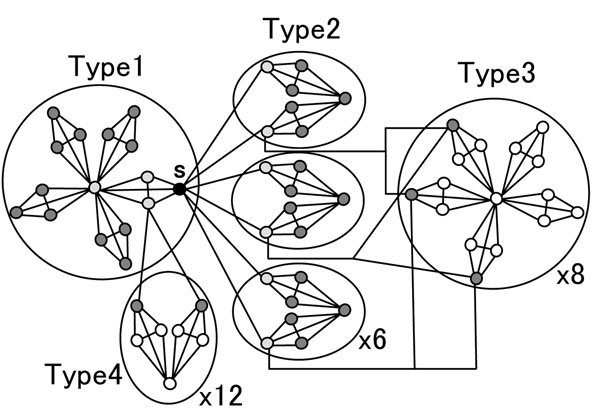
**Two-mismatch subsequences of *s* when *s* is not a code word**. The equivalence classes they belong to are classified into four types.

To refer to all the entries of 2-mismatches, our method requires 27.25 hash keys, which is 25.5% of the number of subsequences with 2 or fewer mismatches. when the length of subsequences is 5. Figure 6 shows the subsequence *s* and its surroundings when *s* is a code word. The number of subsequences of exact 2-mismatches |*N*_2_(*s*)| is 90, and the elements of |*N*_2_(*s*)| are neighbors of *N*_1_(*s*). Each *n* ∈ *N*_2_(*s*) has two neighbors of *N*_1_(*s*) and each *n* belongs to an equivalence class that contains other two elements of *N*_2_(*s*). In other words, 30 equivalence classes are required to cover *N*_2_(*s*). In total, the number of required keys *K*_2_(*s*) is 31. The reference formula is:

In Case 2, *s* is not a code word and Figure 7 shows 2-mismatch sequences of *s*. The equivalence classes 2-mismatch subsequences belong to are classified into four types.

Type 1: 2-mismatch subssequences belong to this type are neighbors of *c*(*s*).

Type 2: for some *t* such that *d_H_*(*t*, *s*) = 1 and *c*(*t*) ≠ *c*(*s*), 2-mismatch subsequences are neighbors of *t* belonging to *c*(*t*).

Type 3: for some *t* such that *d_H_*(*t*, *s*) = 1 and *c*(*t*) ≠ *c*(*s*), they are neighbors of *t* not belonging to *c*(*t*).

Type 4: for some *u* such that *d_H_*(*u*, *s*) = 1 and *c*(*u*) = *c*(*s*), they are neighbors of *u* not belonging to *c*(*s*).

Types 1 and 2 are included in *K*_1_(*s*) and the numbers of subsequences are 12 and 30, respectively. The equivalence classes of Type 3 are the right-most circles in Figure 7. Similar to subsequences of 2-mismatches in Figure 6, three 2-mismatch subsequences belong to each equivalence class. The number of equivalence classes is eight and they includes 24 2-mismatch subsequences. The bottom-left circle in Figure 7 represents Type 4. The relation between of Types 4 and 1 looks similar to that between Types 2 and 1. In fact, Type 4 is a union of *K*_1_(*u*) – *c*(*s*) for over each possible *u*. As the size of *u* is 2, Type 4 has 12 equivalence classes. In total, the number of hash keys to refer to 2-mismatch subsequences is 27 (= 1 + 6 + 8 + 12). The reference formula is as follows:

In the same manner, we can analyze the hash keys *K*_2_(*s*) for the subsequence of length of 21. In this case, the reference formula when *s* = *c*(*s*) becomes:

and the formula when *s* ≠ *c*(*s*) is:

#### Search n-gaps

To align DNA subsequence and a genome, there are three types of gaps. These are gaps in short DNA sequence, gaps in genome sequence, and gaps in both. Our method can reduce the number of hash keys to refer to gaps in short DNA sequences. Given a subsequence with gaps *s*, hash keys to refer to the genome positions are a set of code words of subsequences which with the gaps of *s* are substituted with nucleotides. The expected numbers of hash keys are 3.25 and 3.84 when the length of a subsequence with one gap is 5 and 21 respectively.

Let *s* be a subsequence with one-gap, such as “AA-AA”, and *S* be a set of subsequences for which a gap in *s* is substituted with a nucleotide, in this case the set comprising “AAAAA”, “AACAA”, “AAGAA”, and “AATAA”. To find the genome position that matches *s*, we need to refer to entries that correspond to *S*.

When a subsequence *t* ∈ *S* is a code word, all the subsequences in *S* belong to the same equivalence class. Therefore, by using *c*(*t*) as a hash key, all the entries correspond to gapped subsequence *s* can be referred to from. In this case, the reference formula becomes 4 + 12*x*, where the index number of *x* represents the minimum distance from the four subsequences in *S*.

If no *t* ∈ *S* is a code word, the four subsequences belong to different equivalence classes. Therefore, four hash keys are required and the reference formula is 4 + 60*x*. The proportion for which one member of *S* is a code word is |*s*|/*#* of words in a equivalence class = 4/16 = 1/4, and so the expected number of hash keys is .

In the same way, when the length is 21, the expected number of hash keys is . The reference formulas are shown in Table 2.

Next, we consider a subsequence with two gaps. Let *s* be a subsequence of length 5 with two gaps such as “A-A-A” and *S* be the set of 16 sequences for which the gaps in *s* are replaced with nucleotides. The number of information digits in (5, 3)-PHC is 3, and so one of 16 words in *S* is a code word *t*. The code word *t* is the only code word in *S* because the maximum Hamming distance among words in *S* is 2, which equals the number of gaps, and the minimum Hamming distance between code words is 3. The equivalence class *E*(*t*) includes seven subsequences of *S;* let *U* be the remaining subsequences (*U* = *S* – *N*_1_(*t*)). Because *u* ∈ *U* is not a code word and *d_H_*(*u*, *t*) = 2, the distance between *t* and the code word *c*(*u*) is 3 (*d_H_*(*c*(*u*),*t*) = 3). This implies that *c*(*u*) and *t* differ at a non-gapped position in *t*. Therefore, for all *u*, *v* ∈ *U*, *u* ≠ *v* and *d*(*c*(*u*), *v*) = *d*(*u*, *v*) + 1 ≥ 2. Thus, all the subsequences in *U* belong to different equivalence classes and nine (=|*U*|) hash keys are required to refer to 2-gap subsequences.

For example, if *s*=“A-A-A”, *S* includes a code word *t* =“AAAAA” and *t* can refer to 7 sequences: “AAAAA”, “ACAAA”, “AGAAA”, “ATAAA”, “AAACA”, “AAAGA”, and “AAATA”. The set of remaining subsequences are *U* = {“ACACA”, “ACAGA”, “ACATA”, “AGACA”, “AGAGA”, “AGATA”, “ATACA”, “ATAGA”, “ATAGA” }. Let *u ∈ U* = “ACACA”. The code cord *c*(*u*) is “ACACC” and Hamming distance from the rest of *U* are two or three. Therefore, all subsequences in *U* belong to different equivalence classes.

#### Length of subsequence

**Figure 8:**
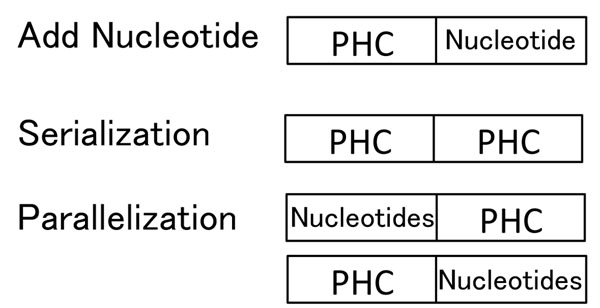
**Three ways to elongate the length of subsequences**.

The code length of the PHC is restricted to 5 or 21 in practice. This is inconvenient. Therefore, we next explain ways to elongate the code length. There are four ways to elongate the code length. The first way is to simply add nucleotides before or/and after the code words. Two other ways are serialization and parallelization of PHCs. The former method serializes more than two PHCs and serialized code words are used as the hash key. The latter one uses more than two hash tables for the parallelization and is a way to utilize the pigeonhole principle where pigeons are mismatches or gaps and holes are the regions without mismatches and gaps. The fourth way is a combination of the three. Figure 8 shows the concepts behind each. In the following, we show a serialized code and a parallelized code, both of length 10.

Let *s* = *s*_1_*s*_2_ be a sequence of length 10 and *s*_1_ and *s*_2_ be the subsequences of length 5. The hash key used in the serialization to store *s* is *c*(*s*_1_)*c*(*s*_2_). In this case, each equivalence class holds 256 subsequences. To refer to the entry of a sequence *s* = *s*_1_*s*_2_ and the 30 1-mismatch sequences, the set of hash keys is:

and the expected number of hash keys is 12.25. To prove this, we consider four cases:

Case 1 *s*_1_ and *s*_2_ are both code words.

Case 2 *s*_1_ is a code word, but *s*_2_ is not.

Case 3 *s*_2_ is a code word, but *s*_1_ is not.

Case 4 neither *s*_1_ nor *s*_2_ is a code word.

In Case 1, Use *s*_1_*s*_2_ as a hash key; this can refer to all the 1-mismatches. The reference formula in this case is the square of the reference formula of length 5, (1 + 15*x*)(1 + 15*x*) = 1 + 30*x* + 225*x*^2^.

In Case 2, we need to consider two subcases based on the position of the 1-mismatch. When the position is in the first half of *s*, the hash key *s*_1_*s*_2_ can refer to all of them. When the position is in the second half, the second half of the hash keys becomes one of the seven words in *K*_1_(*s*_2_). Therefore, a set of hash keys is:

Because *s*_2_ ∈ *K*_1_(*s*_2_), the hash keys in the second subcase include the hash keys in the first subcase. Therefore, the number of hash keys |*K*_1_(*s*)| is 7. The reference formula is:

In Case 3, similar to in Case2, the set of hash keys is:

and the reference formula is same as that of Case 2.

In Case 4, the set of hash keys is the union of {*s*_1_*t*|*t* ∈ *K*_1_(*s*_2_)} and {*ts*_2_|*t* ∈ *K*_1_(*s*_1_)}, which correspond to 1-mismatch in the first half and 1-mismatch in the second half, respectively. Because both of these include *c*(*s*_1_)*c*(*s*_2_), the number of hash keys is 13 (= 7 + 7 – 1). The reference formula is:

The proportions of cases 1 through 4 of 1/256, 15/256, 15/256, and 225/256, respectively, and so the expected number of hash keys is 12.25.

The parallelization requires two hash tables and each subsequence is stored in both hash tables. Therefore, the total size of the hash tables is twice that of serialization. The hash keys are *c*(*s*_1_)*s*_2_: the first haf is PHC, and *s*_1_*c*(*s*_2_): the second half is PHC. Consequently, two types of equivalence classes are used in the parallelization and each equivalence class holds 16 subsequences. The set of hash keys for 1-mismatch sequences is:

where each set corresponds to one of the two hash tables. The expectation number of keys is 13.25.

Let us consider the four cases, which are the same as those for serialization. In case 1, use *s*_1_*s*_2_ as the hash key for two hash tables and all the entries of 1-mismatch are referred to. In this case, the reference formula is (1 + 15*x*) + (1 + 15*x*) – 1 = 1 + 30*x*. As a subsequence *s*_1_*s*_2_ is stored in both hash tables, one is subtracted in the formula. Though the number of hash keys appears to be one, it is used twice. Thus the number of hash keys |*K*_1_(*s*)| is two.

In Case 2, the hash keys are:

The total number hash keys is 8 and the reference formula is:

Case 3 is similar to Case 2. In this case the hash keys are:

In Case 4, The hash keys when the first half includes the mismatch are {*ts*_2_|*t* ∈ *K*_1_(*s*_1_)} and that for the second half are {*s*_1_*t*|*t* ∈ *K*_1_(*s*_2_)}. The number of hash keys is 14 and the reference formula is:

The proportions of the cases are same as for serialization, and the expected number of hash keys is 13.25.

## Discussion

To search genome positions of *n*-mismatches and *n*-gaps with our method, Table 2 shows it also searches some positions of *n*+*α*-mismatch. For example, our method searches not only 1 perfect match and 63 1-mismatch subsequences, but also 210 2-mismatches and 1710 3-mismatches as byproducts 11.1%(= 210/1890) and 4.8%(= 1710/35910) of all 2- and 3-mismatches, respectively. This proportion increases to 46.7%(= 42/90), 20% (= 54/270) when *l* = 5. These byproducts are, in fact, effective. One reason for the current low mapping ratios from DNA sequencers of short reads to a genome is the small number of mismatches and gaps that the employed mapping method can find. Therefore, increasing the numbers of mismatches and gaps will contribute to increasing the mapping ratio and subsequent biological analyses, even if the method is probabilistic.

The increasing demand to map massive amounts of short DNA sequences to genomes is inevitable. Because the number of short sequences is enormous, it is difficult to ensure finding all genome positions of 1-mismatches in a practical computation time. Therefore, faster methods are required and the proposed method is a step in that direction. We have shown that the proposed method can reduce the number of keys necessary to find the genome positions of n-mismatches. The main idea behind the method is to classify the subsequences into equivalence classes using PHC. Because equivalence classes contain multiple subsequences, our method can increase the density of the hash table over those using in the usual method. That is to say, our method can use longer subsequences.

For example, the size of human genome is about 3G bases long. When this is stored it with subsequences of length 21 in usual way, the density of the hash table is 3 × 10^12^/4^21^ ≈ 0.07%. On the other hand, the hash table using our proposed method using (21,18)-PHC, the density is 3 × 10^12^/[# of equivalence classes] = 3 × 10^12^/4^18^ ≈ 4.7%. That is to say, our method can use longer subsequences. The length of subsequence is sensitive to the efficiency of the genome-mapping programs, and the longer the better, for a given density of hash table. Therefore, the proposed method has an advantage from this point of view.

We consider the computation time for code words is far shorter than a hash reference when we describe the effectiveness of the proposed method. In practice, the calculation of the syndrome using the parity-check matrix of the Hamming code is very short, even if on *GF*(2^2^), and so it is easy to calculate the code word from a subsequence. Also, the calculation is small enough to be executed within a CPU cache. On the other hand, the size of hash table is larger than the size of CPU caches. Some exceeds the size of memory because the number of entries is almost equal to the length of the target genome. The hash reference is apparently slower than the calculation of the code word. Therefore, the advantage of reducing the hash references exceeds the disadvantage of additional tasks to calculate code words. With these advantages, our method will help to implement faster genome mapping programs.

## Conclusions

The paper shows perfect hamming code can reduce the number of hash references for hash-based genome mapping. The method encodes subsequences to perfect hamming codes on *GF*(2^2^) and use them as hash keys. It can reduce by about 70% the number of hash keys necessary for searching the genome positions of all 2-mismatches of 21-base-long DNA subsequence. As the amount of data that DNA sequencers generates continues to increase and more accurate genome mappings are required, our method will help to develop faster genome mapping software.

## Competing interests

The authors declare that they have no competing interests.

## Authors' contributions

YT provide the key idea and the mathematical analyses. SS and HM provide valuable help on the topics. All authors discusses the results and commented on the manuscript.

## Supplementary Material

Additional file 1 **DNA sequences and their code words.** All the 5-mer DNA sequences and their code words on 4-ary (5,3)-perfect Hamming codes. Click here for file
